# Computational modelling of emboli travel trajectories in cerebral arteries: influence of microembolic particle size and density

**DOI:** 10.1007/s10237-014-0561-0

**Published:** 2014-03-02

**Authors:** Dario Fabbri, Quan Long, Saroj Das, Michele Pinelli

**Affiliations:** 1 Brunel Institute for Bioengineering, Brunel University, Uxbridge, Middlesex , UB8 3PH UK; 2 Department of Vascular Surgery, Hillingdon Hospital NHS Trust, Hillingdon, Middlesex, UK; 3 Facoltà di Ingegneria, Università di Ferrara, Ferrara, Italy

**Keywords:** Emboli travel trajectories, Cerebral arteries, Circle of Willis, Computational modelling, Ischaemic stroke infarction, Emboli size and density

## Abstract

Ischaemic stroke is responsible for up to 80 % of stroke cases. Prevention of the reoccurrence of ischaemic attack or stroke for patients who survived the first symptoms is the major treatment target. Accurate diagnosis of the emboli source for a specific infarction lesion is very important for a better treatment for the patient. However, due to the complex blood flow patterns in the cerebral arterial network, little is known so far of the embolic particle flow trajectory and its behaviour in such a complex flow field. The present study aims to study the trajectories of embolic particles released from carotid arteries and basilar artery in a cerebral arterial network and the influence of particle size, mass and release location to the particle distributions, by computational modelling. The cerebral arterial network model, which includes major arteries in the circle of Willis and several generations of branches from them, was generated from MRI images. Particles with diameters of 200, 500 and 800 $\upmu \hbox {m}$ and densities of 800, 1,030 and 1,300 $\hbox {kg/m}^{3}$ were released in the vessel’s central and near-wall regions. A fully coupled scheme of particle and blood flow in a computational fluid dynamics software ANASYS CFX 13 was used in the simulations. The results show that heavy particles (density large than blood or a diameter larger than 500 $\upmu \hbox {m}$) normally have small travel speeds in arteries; larger or lighter embolic particles are more likely to travel to large branches in cerebral arteries. In certain cases, all large particles go to the middle cerebral arteries; large particles with higher travel speeds in large arteries are likely to travel at more complex and tortuous trajectories; emboli raised from the basilar artery will only exit the model from branches of basilar artery and posterior cerebral arteries. A modified Circle of Willis configuration can have significant influence on particle distributions. The local branch patterns of internal carotid artery to middle cerebral artery and anterior communicating artery can have large impact on such distributions.

## Introduction

Stroke is the major cause of serious long-term disability and the third most significant cause of death in developed countries, more than 5 million people dying of stroke every year. Survivors of a transient ischaemic attack (TIA) or stroke have an increased risk of another stroke. The majority of them will occur during the first year, which is a major source of increased morbidity and mortality (Adboix and Alio 2010; Tullio and Homma 2002; MacDougall et al. 2009). 70–80 % of strokes are caused by embolic or thrombotic occlusions in the cerebral vessels (or ischaemic stroke).

There are various classification systems for acute ischaemic stroke. The Oxford Community Stroke Project classification (OCSP, also known as the Bamford or Oxford classification) relies primarily on the initial symptoms to describe the influence region of infarction. It can be classified as anterior circulation, posterior circulation infarct and lacunar infarct (infarct possibly caused by the blockage of penetrating arteries). The TOAST (Trial of Org 10172 in Acute Stroke Treatment) classification is based on clinical symptoms and results of further investigations. It denotes five subtypes of ischaemic stroke: (1) thrombosis or embolism due to atherosclerosis of a large artery, (2) embolism of cardiac origin, (3) occlusion of a small blood vessel, (4) other determined aetiology, (5) undetermined aetiology. There is a general agreement that embolism occurs most frequently from arterial and cardiac sources. But in a significant number of cases, the cause remains undetermined, even after exhaustive diagnosis tests (Grotta and Alexandrov 2001; Murtagh and Smalling 2006; Weir 2008).

Emboli resulting from different embolic sources have different compositions and respond differently to treatment. According to a recent AHA/ASA guidance for stroke prevention (Sacco et al. 2006), for patients with non-cardio-embolic ischaemic stroke or TIA (more likely caused by platelet and fibrin- rich thrombi, also called white thrombi), antiplatelet agents rather than oral anticoagulation are recommended to reduce the risk of recurrent stroke and other cardiovascular events, while patients with ischaemic stroke due to cardiogenic embolism (more likely to be a thrombi rich with red blood cells, also called red thrombi) should generally be treated with anticoagulant drugs to prevent recurrence. It is therefore important to know the source of emboli for a specific infarction site diagnosed from medical imaging.

Despite the importance of embolism as a major cause of brain infarction, little is known about the hemodynamic factors that govern the path emboli tend to follow. A natural assumption is that the emboli are distributed in a bifurcation proportionality to flow rate ratios. To have a better understanding of emboli distribution in the brain, many animal studies have been performed over the last 20 years. In one such study (Macdonald et al. 1995), micro particles (normally glass microspheres with diameter $<$ 200 $\upmu \hbox {m}$) were injected into the test animal from carotid arteries. The distributions of spheres of different sizes in brain regions were analysed. It is generally concluded that small particles (diameter $<$ 70 $\upmu \hbox {m}$) can enter penetrating arteries and could therefore produce lacunar infarction. The majority of emboli, however, enter circumferential arteries. In another study (Pollanen and Deck 1990), particles with sizes between 90 and 150 $\upmu \hbox {m}$ were randomly dispersed in leptomeningeal arteries of all vascular regions, while larger ones (150–210 $\upmu \hbox {m}$) were preferentially distributed to the watershed zone. Studies were also carried out on analysing the infarction patterns caused by emboli originating from carotid arteries at different cerebral artery configurations, for example for patients with contralateral ICA stenosis. Tietien et al. (1993) studied the possibility of emboli crossing the territory of a stenotic carotid artery and found that contralateral carotid artery stenosis, like occlusions, will influence the site and size of embolic infarcts and that the “symptomatic” carotid artery cannot always be determined by the site of the cerebral infarct. A more recent study (Rapp et al. 2008) has shown that small emboli (size: 60–100 $\upmu \hbox {m}$) with irregular (thrombus fragments) or needle-like shapes are capable of causing lacunar infarction (block penetrating arteries). The authors concluded that the extent of brain injury from emboli depends upon their composition and shape as well as size.

To date, there have been few experimental studies investigating emboli trajectories in arterial phantoms to improve our understanding of particle behaviour under complex flow condition. Bushi et al. (2005) studied particle trajectories on a single bifurcation phantom for much larger particles (diameters of 0.6–3.2 mm). They found that large emboli (diameter 3.2 mm) would tend to enter the wider vessel more often than expected, given the flow rate ratio, while smaller and mid-sized particles (diameters 0.6–1.6 mm) entered into either the narrow or wider branches without preference; Chung et al. (2010), using a more realistic cerebral arterial phantom which included major cerebral arteries, found that large arteries such as the middle cerebral arteries attracted a higher number of emboli than that would be expected for the flow rate ratio. The particle sizes in their study were 0.2, 0.5 and 1 mm with similar density of blood.

While an experimental study may be able to provide information regarding particle distribution in cerebral arteries, it can be difficult to study the detailed behaviour such as particle speed and travel trajectory. Computational research methods, on the other hand, are able to provide reliable and comprehensive simulations of particle behaviour in complex arterial networks. However, it is difficult to find computational studies on emboli particle trajectories in a realistic cerebral arterial network in the published literature. The present work aims to provide a detailed computational fluid dynamic (CFD) study of embolic particle trajectories in an anatomically realistic model in order to improve our understanding of emboli behaviour in such a complex artery network. The questions that we would like to answer from this study are (a) the influence of particle size, density and release location on their general behaviour such as the distribution in the arterial network and (b) whether it is feasible to predict particle trajectories based on the infarction site and cerebral artery network geometry which can be obtained by conventional scan. The answers to the first question will enhance our general understanding of particle behaviour and to the second question may provide a useful tool on better patient treatment.

## Method

### Reconstruction of the 3D cerebral arterial network

**Fig. 1:**
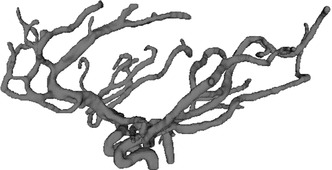
3D surface rendering of the cerebral arterial network

The angiogram of the cerebral arteries was obtained by a 3T MRI system with conventional 3D Time-Of-Flight sequence and voxel size of $0.52\times 0.52\times 0.65\,\hbox {mm}^{3}$. A freeware package “3D SLICER” was used for image processing and the 3D reconstruction. Due to the high quality of MR images, most of the segmentations were performed automatically, while manual segmentation was used for communicating arteries due to their small size. Figure 1 shows a 3D surface rendering example of the cerebral arterial network model generated from “3D SLICER”; it contains all major arteries of the circle of Willis (CoW) and several generations of branches raised from CoW.

### Computational model parameters

**Fig. 2:**
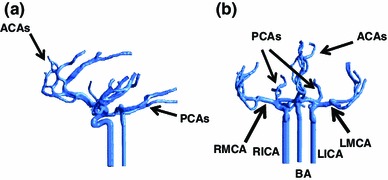
Computation model of the cerebral arterial network with extended inlet vessels. **a** Sagittal view and **b** coronal view

The triangulated surface of the arterial model was input into the ANSYS mesh builder ICEM to generate an unstructured tetrahedral mesh of the fluid domain. To eliminate the influences of inlet flow velocity profile and particle injection velocity on the simulation, the three inlet vessels such as the left and right internal carotid arteries (L_ICA and R_ICA) and the basilar artery (BA) were extended to a length of about 10 times of local diameter as shown in Fig. 2.

Appropriate mesh sensitivity tests for the fluid flow simulation were carried out. Five different meshes were created for this purpose with increasing numbers of elements, as well as prism layers at the vessel wall. The characteristics of the meshes are given in Table 1. A steady state simulation was set for each case, without releasing any embolic particle at the inlets. Six points at the middle cerebral arteries (MCAs), anterior cerebral arteries (ACAs) and posterior cerebral arteries (PCAs) were chosen to compare velocity versus mesh density. Results showed that velocity values at the chosen point were almost identical for the last three cases. Mesh 5 was selected in the study which has a total of 4 million elements and 1 million nodes, 5 layers of prisms at the vessel wall. The mesh quality assessment quantifier “Quality” was selected to present mesh quality which is calculated differently for different element types (Ansys CFX User Manual). For example: “Quality” is calculated as the aspect ratio for the tetra element while the “Determinant” was used for “Quality” for the Quad element. The resulting parameter “Quality” yields a general assessment of the mesh quality by combining a variety of mesh quality criteria. A value of 1 represents the best possible mesh quality and 0 the worst. A mesh with a value of 0.5 or above is considered to be a good quality mesh. In the mesh 5, the overall mesh quality value was 0.85 with the minimum value (for the worst element in the whole fluid domain) above 0.45.

**Table 1:** Meshing details for mesh density sensitivity test

Mesh case	1	2	3	4	5
N of elements	$7 \times 10^{5}$	$1.5 \times 10^{6}$	$2.1 \times 10^{6}$	$3.5 \times 10^{6}$	$3.9 \times 10^{6}$
N of prism layers	0	2	3	4	5
Average quality	0.84	0.79	0.81	0.83	0.85

ANSYS CFX 13.0 was used to solve the governing fluid flow equations. Blood was treated as an incompressible Newtonian fluid with a density of 1,056 $\hbox {kg}/\hbox {m}^{3}$ and a dynamic viscosity of $3.2\times 10^{-3}$ Pa s. The diameters of the vessels in the cerebral arteries are in the range of 1–4 mm. A normal velocity of $0.2\,\hbox {ms}^{-1}$ was set for the blood flow in each inlet vessel. There is no information available of blood flow rate ratio among the exit arteries of the CoW. It can be difficult to use mass flow rate as a boundary condition. In addition, the geometry model includes a similar level of branches at major perfusion regions. Therefore, the flow resistance (or pressure drop) among the main perfusion regions will be similar. Hence, it is safe to say that the pressure boundary condition is suitable for the current simulation set up. In the study, a constant pressure of 0 Pa was used in all exit planes as the outflow boundary condition. The no slip condition was set to model the walls of the cerebral arteries.

Generally, particle tracking simulations can be done by either full or one-way coupling to the background fluid flow. The particle movement will not affect the continuous phase flow field in a one-way coupled model. In a fully coupled model particles exchange momentum with the continuous phase, allowing the continuous flow to affect the particles and vice versa. Our test showed that the velocity contours change slightly at the same location and for the same boundary condition, if injected particles have different size and density for a fully coupled model. But the particles have no influence on the blood flow in one-way coupled cases. Since the influences of particle size and density on particle behaviour are the main objectives of the study, a fully coupled model of particle-fluid interaction is used for all simulations in this study. While this requires much higher computational cost and is also more likely to encounter convergence problems, it does ensure high accuracy in the analysis.

In addition to solving the Navier–Stokes equations for CFD problem, the fully coupled particle-fluid interaction simulation requires to solve additional equations. Considering it is not a normal procedure, the main additional equations for particle tracking are presented here. The momentum transfer for a discrete particle travelling in a continuous fluid medium used in CFX (Ansys CFX User Manual) is based on Newton’s second law, but in a more specific format termed the Basset-Boussinesq-Oseen equation:

1 $$\begin{aligned} m_\mathrm{p} \left( {\hbox {d}U_\mathrm{p}/\hbox {d}t} \right) =F_\mathrm{{D}} +F_\mathrm{{B}} +F_\mathrm{{P}} +F_\mathrm{AM} \end{aligned}$$

where the left hand side represents:

- The particle mass, calculated as: $m_\mathrm{p} =\frac{\pi }{6}\phi _\mathrm{p}^3 \rho _\mathrm{p}$, where $\phi _\mathrm{p}$ and $\rho _\mathrm{p}$ are, respectively, particle diameter and density.
- The particle acceleration: $dU_\mathrm{p}/\hbox {d}t$.

The right hand side of Eq. (1) includes the various forces acting on the particle. The first term characterizes the drag force acting on the particle:

2 $$\begin{aligned} F_\mathrm{{D}} =0.5C_\mathrm{{D}} \rho _\mathrm{{F}} A_\mathrm{{P}} \left| {U_\mathrm{{s}} } \right| U_\mathrm{{s}} \end{aligned}$$

with $C_\mathrm{{D}}$ the drag coefficient,$\rho _\mathrm{{F}}$ the density of the fluid, $A_\mathrm{{P}}$ the effective particle cross section and $U_\mathrm{{s}}$ the slip velocity between the solid particle and the flowing blood. The drag coefficient is modelled as:

3 $$\begin{aligned} C_\mathrm{{D}} =\hbox {max}\left( {\frac{24}{Re_\mathrm{{s}} }\left( {1+0.15Re_\mathrm{{s}}^{0.687} } \right) ,44} \right) \end{aligned}$$

and $Re_\mathrm{{s}}$ of this last equation is the particle relative Reynolds number, defined as:

4 $$\begin{aligned} Re_\mathrm{{s}} =\frac{\rho _\mathrm{{F}} U_\mathrm{{S}} \phi _\mathrm{p} }{\mu _\mathrm{{F}} } \end{aligned}$$

where $\rho _\mathrm{{F}}$ and $\mu _\mathrm{{F}}$ are, respectively, the density and the dynamic viscosity of the blood. $F_\mathrm{B}$ is the buoyancy force due to gravity, given by:

5 $$\begin{aligned} F_\mathrm{B} =\frac{1}{6}\pi \phi _\mathrm{p}^3 \left( {\rho _\mathrm{p} -\rho _\mathrm{{F}} } \right) g \end{aligned}$$

$F_\mathrm{{P}}$ is the pressure gradient force exerted on the particle by the surrounding fluid, and expressed as:

6 $$\begin{aligned} F_\mathrm{{P}} =\frac{1}{6}\pi \phi _\mathrm{p}^3 \nabla p \end{aligned}$$

$F_\mathrm{AM}$ is the force due to the particle’s mass, accelerating the virtual mass of the fluid in the volume occupied by the particles.

Interactions between the particles and the fluid domain’s walls are modelled using a fundamental collision principle that calculates the momentum and kinetic energy of each particle in directions normal and parallel to the wall. Parallel and perpendicular restitution coefficients primarily determine the type of model. A coefficient value of 1.0 implies an ideal elastic collision (momentum and energy are conserved), whereas any value less than one indicates a nonelastic collision (momentum but not energy is conserved), a value of zero resulting in particle deposition on the wall. In this study, both the perpendicular and the parallel coefficients were 1.0, and the arterial wall was modelled as a smooth surface without any surface roughness (Basciano et al. 2010). The coefficient value of 1.0 was also assigned (as a default value in CFX) for particle–particle collision simulation.

**Fig. 3:**
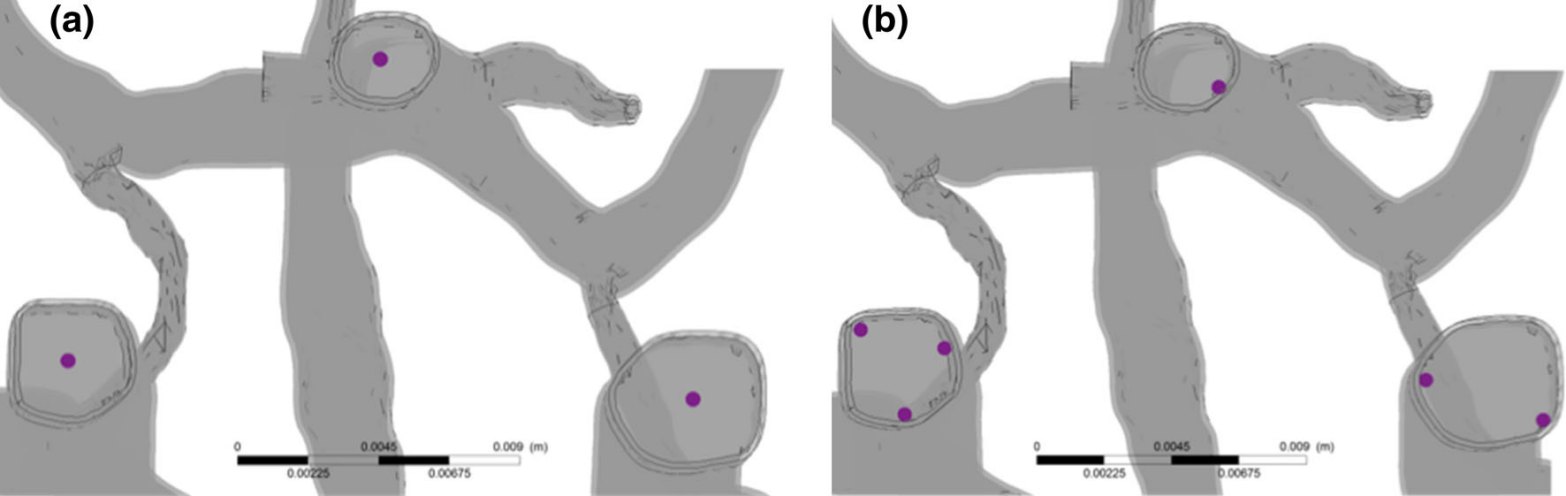
Region selected to release particles in the fully coupled model. Three locations are in the Core flow region (**a**) and 6 in the Near-wall flow region (**b**)

A high accuracy scheme (CFX default) was used to discretize the advection term of the Navier–Stokes equations; An absolute residual of $10^{-4}$ and 20 loops per time step were used as convergence criteria for all variables. A transient analysis (but with constant boundary conditions) was used to simulate flow through the arteries, with a time step of 0.01 s and a total simulation time of approximately 5 s, which means eventually 500 time steps were calculated to give enough time for most particles to leave the system. It is quite likely that some particles may get trapped in regions of slow moving flow or even recirculation zones in the model. Since the main objective of the study is to simulate the particle distribution and general behaviour in the cerebral arteries, only major features of particle behaviour are presented, with particles trapped in recirculation zones being generally ignored in the analysis of results, Solid particles were released in two different regions of the inflow planes, such as the core and the near-wall flow region as shown in Fig. 3. For the core release cases, the central point in each inlet plane was chosen with one particle to be released at each time step for each inlet. A total of three particles are released per time step for the core release cases. Similarly, six different positions were chosen in the near-wall region: three in the L_ICA, two in the R_ICA, and one in the BA. Hence, in total 6 particles were released in the near-wall release cases for each time step. Approximately 300 particles were released in the system at the first phase of simulation. The simulation was then stopped and restarted with the same conditions but without particle release to model the particle transport of the second phase. Embolic particles were injected in each site with a normal velocity of $20 ~\hbox {cm}/\hbox {s}$, the same as the blood flow. A particle will travel at a similar speed to the surrounding flow before it reaches the first bend of the inlet vessel.

Eighteen simulations were carried out to study the effects of size, density and initial condition of the particles on their behaviour. The case and group name with their diameter and density are given in Table 2. Three different diameters were set to the values of 200, 500 and $800 \upmu \hbox {m}$ to represent micro particles (200 $\upmu$m), and different medium sized particles. The particle density selection is based on a comparison with blood density. One with smaller density than blood will represent red emboli which normally have a large proportion of closely packed erythrocytes. The red emboli are generally large, softer and likely to be lighter, and more originated from the cardiac source. White emboli, on the other hand, are normally smaller, harder and densely packed with fibrin and are likely to be denser than blood. White emboli are more frequently found if a stroke is caused by carotid artery occlusion of embolic origin. Despite the importance of emboli particle physical property, no measured density values of different embolic particles are available in the literature. To assess the impact of density on particle trajectory, we allowed for a slightly larger range from $200\,\hbox {kg/m}^{3}$ below to $200\,\hbox {kg/m}^{3}$ above the blood density. This gave 800, 1056 and 1,300 $\hbox {kg}/\hbox {m}^{3}$, respectively, less than, similar to and greater than that of blood. The cases are divided into three groups according to their diameter, as shown in Table 2. For each case, two basic simulations were carried out for core (or central) and near-wall particle release.

**Table 2:** Study case name definition grouped with respect to diameter, density and mass

Study case	A1	A2	A3	B1	B2	B3	C1	C2	C3
Diameter $(\upmu \hbox {m})$	200	200	200	500	500	500	800	800	800
Density $(\hbox {kg/m}^{3})$	800	1,050	1,300	800	1,050	1,300	800	1,050	1,300
Mass (mg)	0.0268	0.0352	0.0435	0.419	0.55	0.68	1.715	2.251	2.787

Six further simulations were also performed for each case, as follows:

- two for a different outflow boundary condition: core and near-wall release of simulation with fixed mass flow rates on all but one efferent arteries of the CoW;
- four for two modified CoW configurations again with both core and near-wall released particles.

## Results

### Cerebral artery network

Figure 2 shows the cerebral arterial network from two angles of view. Together they represent a typical CoW configuration for a normal human. It can be noticed that anterior arteries are generally located in the sagittal plane (around the symmetrical plane of the left and right brain). Approximately two generations of arterial branches from the main ACA are accommodated within the model, forming a small network. Both left and right MCAs and their branching vessels were located in curved surfaces as shown in Fig. 2b. Again, a few branches from the main MCAs were included in the model. The PCAs mainly pointed in the posterior direction. Although not shown in Fig. 2 the communicating arteries were, in fact, included in the CoW model.

### Flow divisions

The predicted set of solutions for the flow rate ratio among the exit arteries is shown in Table 3: this set gives a background reference for the flow system. The flow rates in the feeding arteries, imposed as simulation boundary conditions, are also listed in the Table. It can be seen that the total blood flow rate in both MCAs is about 57 %. As for the remaining arteries, the ACAs have a slightly reduced flow rate compared with the total flow rate of the small branches of the BAs and PCAs. This result is consistent with similar levels found in recent clinical measurements in which the flow rate ratio for ACA:MCA:PCA was found to be 20:60:20, respectively, for a typical circle of Willis perfusion (Tanaka et al. 2006). From Table 3 it can also be seen that blood from the feeding carotid arteries will provide flow in the MCAs, ACAs and part of the PCAs. Flow entering from the BA will normally go to the small branch vessels in the BA and PCAs. Since constant mean velocities were used as the inlet flow boundary conditions, because and the diameter of the R_ICA is slightly larger than the L_ICA, the flow rate in the R_ICA is slightly higher than the L_ICA. The flow rate ratio from the feeding arteries is R_ICA:L_ICA:BA = 45:33:20, compared with the ratio 40:40:20 measured by Tanaka et al. Collateral flow rates are minimal through the communicating arteries.

**Table 3:** Predicted flow rate distributions in major arteries. (unit %)

Outlets	Right_MCA	%	Left_MCA	%	ACA	%	BA+PCA	%
Branching vessels	R_MCA_O1	10.06	L_MCA_O1	10.35	ACA_O1	3.58	BAS_O1	7.37
	R_MCA_O2	1.77	L_MCA_O2	7.78	ACA_O2	5.95	BAS_O2	3.90
	R_MCA_O3	3.95	L_MCA_O3	3.62	ACA_O3	3.77	L_PCA_O	7.83
	R_MCA_O4	4.49	L_MCA_O4	4.68	ACA_O4	1.86	R_PCA_O1	2.96
	R_MCA_O5	6.36			ACA_O5	1.13	R_PCA_O2	2.34
	R_MCA_O6	4.06			ACA_O6	2.11		
Total		30.70		26.43		18.41		24.46
Inlets	R_ICA	45.95	L_ICA	33.48			BA	20.57

### Particle behaviour: average travel speed and resident time

**Fig. 4:**
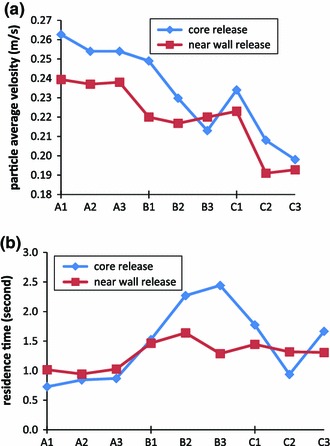
Influence of particle mass on their motion in cerebral arteries. Particles were released at core location (*blue lines*) and near-wall locations (*red lines*): **a** Average residence times of particles; **b** average velocity of particles travelling during their path. On x-axis: A, B, C representing particle density of 800, 1,050, 1,300 $\hbox {kg}/\hbox {m}^{3}$; number *1,2,3* representing particle diameter of 200, 500 and 800 $\upmu \hbox {m}$ as defined in Table 2. (Color figure online)

Figure 4 demonstrates the effects of differing mass on the general behaviour of particles in the cerebral arteries. The first general feature that can be seen is that for particles of higher mass the travelling speed decreases, for both core and near-wall released particles (Fig. 4a): this is as one would anticipate. The particle travelling time in the model, before exiting from the system, increases for core released particles with small and medium diameters but decreases for larger particles. For near-wall released particles, the travelling time curve is more stable, with a slight increase for particles ranging in size from small to medium and large (Fig. 4b).

**Fig. 5:**
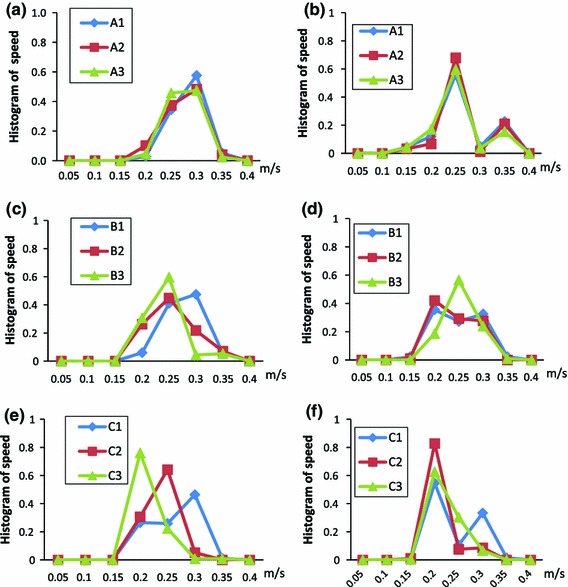
Histogram of particle travel average speed distribution for all particles released in the system for the 9 different particle masses. Panels on the *left* (**a**, **c**, **e**) are core release cases while panels on the *right* (**b**, **d**, **f**) are near-wall release cases. In *curve labels*: *A*, *B*, *C* representing particle density of 800, 1,050, 1,300 $\hbox {kg/m}^{3}$; number *1,2,3* representing particle diameter of 200, 500 and 800 $\upmu \hbox {m}$ as defined in Table 2

Since particles adopting different travel routes may have significantly differing speeds and residence times, detailed data for distributions of particle travel parameters for all released particles given in a histogram manner may provide a more accurate description of particle behaviour than giving a general average. Figure 5 provides such data, giving further details of particle travel speed distribution as a normalized histogram for all particles released (usually more than 300) in the system. It can be seen that for the core released cases (left panes in Fig. 5), the histogram generally appears as a normal distribution of speed values with the central velocity gradually separating for the different cases within the same group. Groups A, B, and C represent particles with densities of 800, 1,050 and 1,300 $\hbox {kg/m}^{3}$, respectively, and cases 1, 2 and 3 have particle diameters of 200, 500 and 800 $\upmu \hbox {m}$, respectively. For core release cases in group A (Fig. 5a), the velocity distributions are very similar for particles of different diameter, with the peak value at about 0.3 m/s. This agreement changed slightly for the cases in group B, in which the common peak value moved to 0.25 m/s for the denser particles. The three distribution curves separated completely for the cases of group C with the peak values being 0.3, 0.25 and 0.2 m/s: this clearly shows that a denser particle is more likely to travel at a slower speed than a less dense one. The results also demonstrate that the peak velocity values are almost the same for small density particles (i.e. for all cases in group A where the density is less than that of blood), irrespective of their size.

For the near-wall release cases (right panels in Fig. 5), the histogram curves are significantly different from their core release counterparts. A clear “M” shaped curve can be seen in Fig. 5b for the three cases. Convergence of the curves for all cases in group A, as evidenced in Fig. 5b reflects the similar convergence for the core release cases in group A (shown in Fig. 5a). The M shaped histogram curve also holds for the smaller particles in group B (Fig. 5d), but disappears for the 800 $\upmu$ particles. In group C (Fig. 5 f), the M shaped curve holds only for case C1. F or both cases C2 and C3 a single peak histogram curve appears with the peak velocity value reducing slightly from case C2 to C3.

**Fig. 6:**
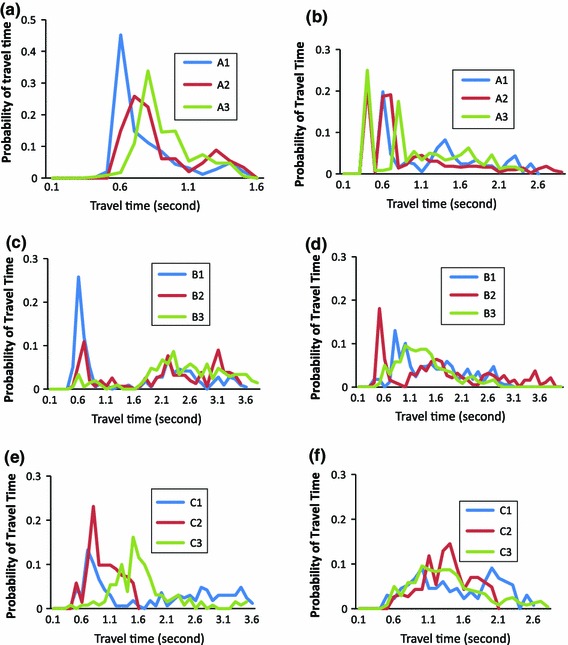
Histogram of particle travel time probability for the nine test groups in core release (*left panels*) and near-wall release cases (*right panels*). In *curve labels*: *A*, *B*, *C* representing particle density of 800, 1,050, 1,300 $\hbox {kg/m}^{3}$; number *1,2,3* representing particle diameter of 200, 500 and 800 $\upmu \hbox {m}$ as defined in Table 2

Histogram results for particle travelling times are shown in Fig. 6. They have a much wider distribution with the curves being much more irregular than those for the velocity distributions. For the core release cases (Fig. 6 left panels), it can be seen that a main peak of the histogram appears for cases A and B between the travel times of 0.5 and 1.0 s. Also, for the cases in group A (Fig. 6a) a small second peak between times 1.3–1.6 s can be identified, while for the group B cases (Fig. 6c) the second peak is at 2.3 s with a long tail until 3.6 s. The peaks of the travel time distribution for the group C cases (Fig. 6e) are separated slightly by which denser particles (case C3) have the longer peak time of 1.5 s with others around 0.7 and 0.8 s for C1 and C2, respectively. Almost all particles have exited for the C2 case after 1.5 s. For the C1 and C3 cases, only very few particles remain in the system beyond 3.6 s.

For the near-wall release cases (Fig. 6 right panels), A1 and A3 (Fig. 6b) have few particles exiting the system within 0.4 s of their release. A few main travel time peaks remain in the distribution curves for the group A cases followed by a long tail until about 3 s. The distribution curves of B1 and B2 (Fig. 6d) show one main peak followed by a few smaller peaks. Although the small peaks are located at similar time points, the main peaks are at 0.8 and 0.5 s for B1 and B2, respectively. The main peak for case B3 is largely delayed to 1.3 s with the curve being very wide with no obvious secondary peak following. The travel time distribution curves for the group C cases (Fig. 6f) are very similar showing a main peak with a wide curve spread from 0.5 to 2.6 s.

### Particle distributions

**Fig. 7:**
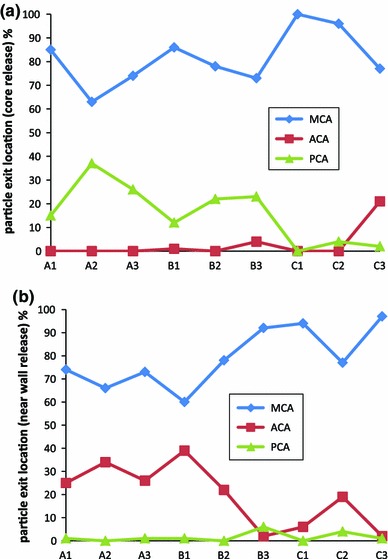
Distributions of particle in the cerebral arteries released from **a** the central location and **b** the near-wall locations of both ICAs, for different groups of particles. On x-axis: *A*, *B*, *C* representing particle density of 800, 1,050, 1,300 $\hbox {kg/m}^{3}$; number *1,2,3* representing particle diameter of 200, 500 and 800 $\upmu$ as defined in Table 2

Figure 7 shows the distributions of particles in the cerebral arterial network for particles released from both ICAs. For all cases, the majority of particles ($>$60 %) flow into the MCAs. When analysing the MCA results, the average number of particles flowing into the MCAs for all cases is 81 % for core and 79 % for near-wall released particles. It is also found that for the core release cases, particles with the smallest density of 800 kg/m$^{3}$, that is, less than blood, (cases A1, B1, C1) generally result in a higher rate of particles entering the MCAs (average = 90%), while for particles with a density of 1,300 $\hbox {kg/m}^{3}$ that is, higher than blood, (cases A3, B3, C3,) the average is 74%. However, this trend disappears for the near wall release cases, where the likelihood of particles entering the MCAs increases with increased particle diameters.

Another feature is that particles hardly enter the ACA for those released from the core flow of ICAs, except for the most heavy particles (case C3). Indeed particles stop entering the PCA when their size becomes large (for cases C1, C2, C3). For the near-wall release cases, on the other hand, almost no particle enters the PCA if they are released from ICAs.

It is interesting to notice that 100 % of particles released from the BA enter the PCAs.

### Particle path tortuosity

Particle path tortuosity is another parameter of interest, in describing embolic particle motion in cerebral arterial networks. In the study, the particle path tortuosity is measured by comparing the difference between the actual particle path length of a specific particle and the sum of the length of the vessel’s central line. The quantitative path tortuosity is calculated as: (particle’s true path length—route vessel centre line length)/centre line length, and is presented as a percentage. The larger this value is, the more tortuous the path will be. The value is zero if a particle always travels on the vessel’s centre line which is defined as the shortest path.

**Fig. 8:**
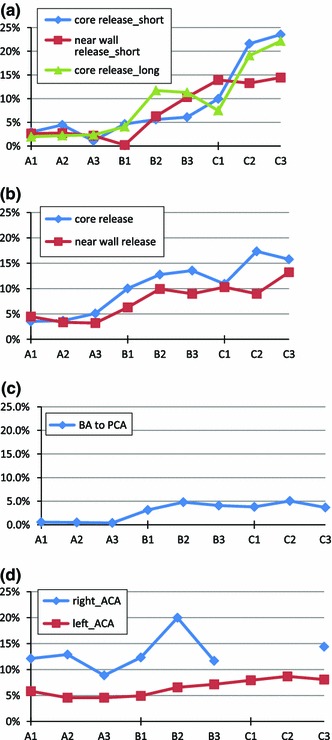
Tortuosity of particle travelling path in different vessels. **a** Right ICA to right MCA_1 (short path) and right MCA_2 (long path); **b** left ICA to left MCA; **c** BA to left PCA core release only; **d** right ICA to right ACA (*diamond sign*) and left ICA to left ACA (*rectangular sign*), core release only; Due to lack of particle exit to the chosen route for near-wall release, the results of near-wall release cases do not present here for some cases

From Fig. 8a–c, the path taken by particles flowing into the MCAs and PCAs becomes more tortuous and complex for medium and large size particles for both core and near-wall release. For small particles (group A cases with a diameter of 200 $\upmu$), the tortuosity is minor, with the particle path being slightly longer than the length of the vessel’s central line.

The left vessels, in the near-wall release cases, normally have a smaller tortuosity than in the core release cases. Due to the asymmetry of the arterial network and slight difference in mass flow rate, particles on the right side of the cerebral arteries have more complex paths for core released large-sized particles. The two particle path routes from the R_ICA to the R_MCAs represent particles that exit from the two R_MCA branches. It is also interesting to notice that the particle path tortuosity is very similar for both long and short routes to the right MCAs (Fig. 8a). The near-wall release cases for the right MCA routes have a smaller tortuosity than the core release cases for large particles, but an even larger tortuosity for medium sized particles.

The tortuosity of the particle path from the BA to the PCA is generally small (Fig. 8c, $<$5 %). In this case, the length of particle path is almost the same as the vessel’s central line for small particles and slightly larger for medium and large particles. According to Fig. 8d, particle path tortuosity is generally large for particles travelling through the right ACA, with no clear trend evident of the variation in its value for different particle sizes and weights. For cases C1 and C2, since there is no particle going into the right ACA, a zero tortuosity ensues. The path tortuosity of a particle travelling from the left ICA to the left ACA appears to remain in a narrow band of change within all tested cases. Their values are smaller than for the particles in the right side of the cerebral arterial network. It should be noted that due to a lack of particles exiting to the chosen routes for the near-wall release, no results for these are presented here.

### Particle distribution for CoW models with changed outflow boundary or CoW configurations

The treatment of outflow boundary conditions in the above simulations is straightforward, and the simulations converge easily. However, there is no direct control of the exact flow rate in the exit vessels. An additional simulation named M1 was designed to test the outflow boundary condition setting and its impact on particle distribution. In the M1 simulation, fixed mass flow rates were defined on all but one exit vessels. A pressure boundary was defined on the exit boundary plane of an artery in the group of L_MCA vessels to avoid over defining the boundary condition and to ensure that the mass conservation condition is met.

It is also well known that the configuration of the CoW can vary significantly in human populations. To assess the impact of the CoW configuration on particle behaviour, based on the model above two CoW models were generated, by artificial removal of certain communicating arteries in the CoW. Model M2a is obtained by removing the left posterior communicating artery (L_PCoA) from the CoW; model M2b by removing the right anterior communicating artery (R_ACoA) from the original CoW. Mesh densities for the new models were similar to the original one. Inflow and outflow boundary conditions were also the same as with the original simulation. Due to the pressure outflow boundary, the flow rate ratio among the exit vessels changed with the different CoW configurations. The particles of diameter 500 $\upmu$m and density 1,050 $\hbox {kg/m}^{3}$ were used in this part of study (case B2 in previous sections).

Table 4(a)–(c) presents the particle distribution results for M1, M2a, M2b, respectively. To facilitate appreciation of the comparison, results obtained in the original simulation (case B2 above) are also presented in Table 4(d) in the same format. One common feature for every case is that for core release, particles released from R_ICA will exit only from R_MCA branches (100 %) for all of the cases. Although not presented here, all particles released from BA exit the model from posterior arteries.

**Table 4:** Particle and flow rate distribution for different models

	ACAs	MCAs	BA+PCA
(a) Particle distribution in Model M1 with fixed mass flow boundary condition			
* Core*			
R_ICA	0	100	0
L_ICA	24.3	75.7	0
* N_W*			
R_ICA	2.1	97.8	0
L_ICA	33.9	66.1	0
(b) Particle distribution in model M2a (removal of L_PCoA)			
* Core*			
R_ICA	0	100	0
L_ICA	9	91	0
* N_W*			
R_ICA	20.0	80.0	0
L_ICA	21.1	79.9	0
(c) Particle distribution in model M2b (removal of R_ACoA)			
* Core*			
R_ICA	0	100	0
L_ICA	15.2	67.1	17.70
* N_W*			
R_ICA	0	79.1	20.80
L_ICA	90.1	9.5	0
(d) Particle distribution in model B2 (original model)			
* Core*			
R_ICA	0	100	0
L_ICA	0	52.6	47.4
* N_W*			
R_ICA	3.1	96.1	0
L_ICA	37.8	62.2	0
(e) Flow rate ratio for M1			
Right	24.4	75	0.54
Left	25.4	75	$-$0.4
Overall	20	60	20
(f) Flow rate ratio for M2a			
Right	26.70	67	6
Left	19.10	80.90	0
Overall	18.67	57.89	23.40
(g) Flow rate ratio for M2b			
Right	0	90.6	9
Left	33	65.6	1.30
Overall	11.05	63.61	25.32
(h) Flow rate ratio for the original model B2			
Right	27.30	66.80	5.90
Left	17.50	79	3.50
Overall	18.41	57.13	24.46

Results are presented in percentage. For Panels a–d: Row: particle release location, Column: particle exit location. Core: core release, N_W: near-wall release. Panels e–h: mass flow rate ratios for the models on the left, right side of the CoW and the overall ratio for the three perfusion territories. Flow from BA is included in the overall value

Comparing Table 4(a) and (d), it may be noticed that the particle distribution from M1 is very similar to those in B2 for near-wall released cases. There is, however, a large difference for core release in the left side. Instead of exiting from the posterior region, as in the B2 case, now 24 % of particles will leave via the ACA, with no particles at all traversing the posterior region. This variation may be caused by the slight change in flow rate ratio between ACAs and BAs. In the B2 case, about 3.5 % of the flow from LICA entered the posterior region via L_PCoA (Table 4(h)). However, the flow in L_PCoA changed direction from posterior to L_MCA (Table 4(e)).

Comparing simulations M2a and M2b with modified CoW, there was a reduced influence on the particle distribution by removing L_PCoA (M2a). It may be noticed that the removal of L_PCoA has very limited influence on the flow rate distribution for different territories on the right side of the CoW, as shown in Table 4(f) and (h). However, in the near-wall release case, the particle distributions on the right side were affected, with an increased number of particles entering the ACAs.

For the M2a model, for all release locations, no particle at all exits from the posterior region. At the left side, more particles exit from the MCAs, although slightly more flow enters the ACAs in case M2a compared with B2.

Comparing cases B2 and M2b, the absence of R_ACoA has a significant influence on both the flow balance in the CoW on both sides, and on the overall flow rate ratio (Fig. 4g, h). Also, the flow rate from L_ICA to L_ACAs increases from 17 % for B2 to 33 % for M2b. This change in the flow rate results in 90 % of the particles being washed out from ACA when they were released from the near-wall in L_ICA. Due to the missing R_ACoA, about 20 % of particles went into the posterior region on the right side near-wall release case, compared with the zero value for all other simulation cases in this group.

**Fig. 9:**
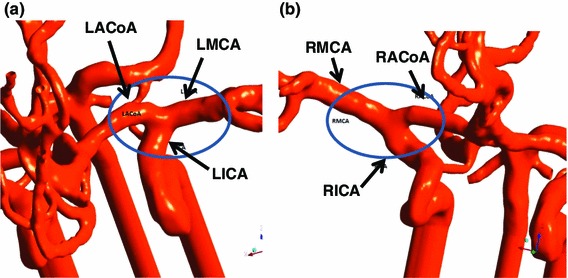
Local geometry of CoW branches. **a** *left side*, shows a *T* junction shape bifurcation from LICA to LMCA and LACoA; **b** *right side*, shows a *Y shape* branch but flow is from RICA to RMCA and RACoA

Comparing all four cases, it should also be noted that particle distributions among the three territories change the most for left side core release cases. In another words, particle distribution is more sensitive to the flow rate on the left side of the CoW. The local geometry of the left side arteries in the CoW may be the reason. Closer inspection reveals that the joint of LICA (Fig. 9a), L_MCA and L_ACA resembles a T junction: this geometry results in more particles entering the L_ACA. However, on the right side, the local geometry is totally different, as is shown in Fig. 9b. In the right side branching geometry, a large proportion of particles will go to R_MCA regardless of the flow rate ratio for the efferent vessels. Core release particles with high energy will have only a very limited likelihood of travel to vessels other than the R_MCA.

## Discussion

Stroke still remains the third most significant killer in developed countries. However, a large percentage of the patients who suffer a first TIA or stroke will survive the first attack. It is the recurrence of the symptoms and consequent strokes that cause the majority of deaths or disabilities. The prevention of recurrence of TIA or a major stroke is largely dependent on a patient’s long-term treatment. Knowing the exact source of emboli has been identified as the key in conducting treatment (Sacco et al. 2006). Due to the complex nature of blood flow from the major feeding arteries to the small arteries in the cerebral arterial network, accurate tracking of a specific particle from its source to the infarction site is a difficult task. The problem of pinpointing a potential emboli source from a known infarction site, by medical diagnosis, is actually an inverse problem which makes the accuracy of such diagnosis impossible. Currently, clinicians base their diagnosis on patient history and experience of cardiovascular problems. However, there are still a large number of cases where the emboli sources cannot be diagnosed. The present study attempts to provide some more general emboli information by employing a comprehensive model to address detailed particle behaviour in the complex cerebral arterial network. With more studies of this kind in the future, clinicians may be able to diagnose emboli sources and provide better care to the patients in preventing the recurrence of strokes.

As suggested by previous experimental studies, emboli particles do not normally flow into a branching vessel based on the basis of the flow rate ratio. Large particles have been shown to have a larger possibility of entering a branching vessel which has a large diameter. The present study also demonstrates this feature of particle behaviour. In addition our study provides more comprehensive comparisons, by addressing the effects of size and density. Smaller particles released from ICAs (group A with diameter $<200$ $\upmu \hbox {m}$) have a slightly reduced possibility of entering the MCAs in the near-wall release cases compared with the larger ones. However, that possibility is still higher than is suggested by the flow rate ratio which is about 57 % for both, core and near-wall release cases. The second effect is that of particle density. Those with a density less than that of blood will be more likely to travel to the MCAs (average $>$90 %), regardless of their size. A result of interest in this group of simulations, is that for particles released from the central lumen region of ICAs, hardly any will travel through the ACA. The situation changes totally for particles released from the near-wall region of ICAs: it was found that no particle enters the PCA. We cannot fully explain this result since only a few locations were chosen for particle release.

Another main result from this study is that large embolic particles will travel at a slower speed than both their smaller counterparts and the surrounding blood. Detailed examination of the particle average speed distribution histograms has shown that the variation occurs only in the cases of groups B and C. For core released particles, the central speed value shifts from 0.25 to 0.2 m/s for large particles in the histogram. In addition, for the near-wall release cases, the fast moving peak disappears in the histogram for large particles. However, these changes are not entirely dependent on the particle mass. A large particle with small density (such as the case of red emboli) may have a higher travel speed such as in cases B1 and C: this indicates that particle density and mass may jointly influence particle speed. When a particle is very small, as for example in the group A cases, density is not significant in influencing its travel speed.

There are several possible reasons for a large particle to move more slowly. They are (a) the particle travels in a slow moving fluid region, namely near-wall regions; (b) the large mass inhibits acceleration; (c) a large drag force acting on the particle. A large particle which also has a high density will, due to its large mass, be more likely to have a small acceleration and combined with a large drag force (due to its size) means its motion is reduced. However, for a large particle with a small density (less than that of blood) such as in C1, despite its total mass being larger than for a smaller particle with high density (for example B3) and hence still have a high drag coefficient $\hbox {C}_{\mathrm{D}}$ by virtue of its size will be subject to a buoyancy force which outweighs those effects: this means that such a particle may still stay in the fast motion region in a tube flow.

The particle trajectory tortuosity results indicate that a particle with large momentum (due to a large mass) travels at a higher speed (in the central region or in large arteries in the MCA) and generally has a more complex route. This may be due to the fact that this kind of particle is more likely to bounce from the arterial wall and as a result change its path. It is worth pointing out that a particle travelling in the near-wall region will not necessarily produce higher tortuosity. It is also of interest to note that particle path tortuosity is generally small for particles going to the ACA, despite their much longer and complex journeys than particles exiting from MCAs. Due to the reasonably straight and simple branching structure in the BA and PCA vessels, particle path tortuosity is small for particles travelling in these vessels.

In general, the clinical impacts of particle trajectory tortuosity are not clear. One aspect of behaviour that may be expected is that for a real embolus with a more irregular shape (not spherical as assumed in the present study), a more complex travel path means increased tumbling of the particle. This may introduce an additional drag force causing the embolus to slow down and to aid it to dissolve.

Apart from the study on the impacts of particle size and density on the particle distribution, simulations were also carried out on different outflow boundary conditions and modified CoW configurations. Although the outflow boundary condition, defined as a fixed mass flow rate, provides better control of flow rate ratio in the CFD simulation, the particle distribution pattern does not change significantly. The modifications of the CoW configuration by artificially removing some communicating arteries, however, do produce significant changes in the particle distribution. By analysing the morphological difference between the left and right side of the CoW, it can be seen that the sensitivity of particle distribution to the changes in CoW configuration can be greatly influenced by the local vessel branching patterns. Further studies are needed on the particle distribution with a wider range of CoW models.

In the present study, particles were released from the same points in the central or near-wall locations with a rate of one particle per time step. Ideally, all of the particles released from the same location should follow the same trajectory since the other flow conditions remain the same during the simulation. But the complex geometry of the major branches of the cerebral circle of Willis results in a flow pattern of extreme complexity. It is the mixing of the particles and the blood itself, flowing together in such irregular flow regions, which dominates both the particle trajectory and the final particle destination. A very slight variation in the impact of the fluid flow on the particle, for example as a result of particle-particle interaction, may cause the particle to travel in a totally different route. However, at this level of such variations numerical simulation uncertainties become more significant, meaning that it can become very difficult to predict reliably the exact trajectory for a specific particle. In other words, it is not feasible to pinpoint accurately the source location for a specific embolus particle found at a stroke site if the simulation is based only on the vessel network geometry. None the less, we could provide an estimate of the originating location of the embolus, which could be valuable in improving diagnostic procedures.

Relevant to the above discussion were the repeatability tests carried out on predictions of the particle paths. Simulations for some cases were repeated with the same conditions so that the influence of numerical uncertainties would be similar for each simulation run. The results showed that although it is difficult to predict the trajectory for a specific particle, the above mentioned probability of particle distribution in fact repeated very well, together with all other parameters. We also tested the repeatability of a single particle trajectory by releasing only two particles from the same central location at different times, instead of one particle from one location for each consequent time step as in the presented simulations. The particle trajectories were almost identical for the two particles. This partially demonstrated that the different trajectories for particles released from the same location may be due to particle-particle interaction in the flow, as discussed above.

There were limitations on the study, a major one being the high computer usage. A single case study required about 3 days CPU time on a Quad-core PC workstation. This was due to the time required for a particle to exit the arterial network (up to 5 s of particle travel time), relative to the fine time step resolution used (0.01 second). Therefore, the overall study had to be designed in a simplified manner, that is, to assume steady flow with only central and near-wall particle release locations. However, other parameters will also influence particle trajectory results. These include: particle release time in a pulsatile flow situation; varied release locations; and arterial network model geometry variations in terms of artery sizes of the MCAs, ACAs and PCAs. In a fully coupled particle simulation, coefficient values were defined to simulate particle–particle and particle–wall collision effects. In this study, a coefficient value of “1” was used to represent a fully elastic collision. If the value was reduced to 0.8, we found it significantly influenced by the particle distribution. We conclude therefore that more studies are needed to investigate further of the sensitivity of the results to the coefficient value. We would also like to point out that the inlet flow rate used in our study was 210 ml/min. This is at the lower end of the clinical data range (with a typical value of 250 ml/min); hence, the predicted blood velocity values as well as particle speeds may also be at the lower end of typical clinical conditions. Since the main purpose of the current study is to gain a general understanding of particle travel behaviour in a complex arterial network including the impact of particle size, weight and release locations (both, in terms of fast and slow flow regions) to the particle trajectory, it may be concluded that the results obtained from the simplified model are still very useful.

## Conclusion

Despite the complex nature of the problem, this study has reached the following conclusions: Heavy particles (density larger than blood with a diameter larger than 0.5 mm) normally have small travel speeds in arteries; Large or lighter embolic particles are more likely to travel to large branches in cerebral arteries. In certain cases, 100 % of the particles go to the MCA; Large particles with higher travel speeds in large arteries are likely to follow more complex and tortuous trajectories; Emboli released from the BA will only exit the model from the branches of BA and PCAs; Modified CoW configuration can have significant influence on the particle distributions. The local branch patterns of ICA to MCA and ACoA can have a large impact on the particle distributions.
